# Aspirin inhibits proliferation and promotes differentiation of neuroblastoma cells via p21^Waf1^ protein up‐regulation and Rb1 pathway modulation

**DOI:** 10.1111/jcmm.14610

**Published:** 2019-08-20

**Authors:** Giacomo Pozzoli, Giovanna Petrucci, Pierluigi Navarra, Hany E. Marei, Carlo Cenciarelli

**Affiliations:** ^1^ Institute of Pharmacology Università Cattolica del Sacro Cuore Rome Italy; ^2^ Pharmacology Unit Fondazione Policlinico Universitario A. Gemelli IRCCS Rome Italy; ^3^ Department of Cytology and Histology, Faculty of Veterinary Medicine Mansoura University Mansoura Egypt; ^4^ Institute of Translational Pharmacology (IFT) National Research Council (CNR) Rome Italy

**Keywords:** acetylsalicylic acid, cell cycle, neuroblastoma, neuronal differentiation, Rb1

## Abstract

Several clinical and experimental studies have demonstrated that regular use of aspirin (acetylsalicylic acid, ASA) correlates with a reduced risk of cancer and that the drug exerts direct anti‐tumour effects. We have previously reported that ASA inhibits proliferation of human glioblastoma multiforme‐derived cancer stem cells. In the present study, we analysed the effects of ASA on nervous system‐derived cancer cells, using the SK‐N‐SH (N) human neuroblastoma cell line as an experimental model. ASA treatment of SK‐N‐SH (N) dramatically reduced cell proliferation and motility, and induced neuronal‐like differentiation, indicated by the appearance of the neuronal differentiation marker tyrosine hydroxylase (TH) after 5 days. ASA did not affect cell viability, but caused a time‐dependent accumulation of cells in the G_0_/G_1_ phase of the cell cycle, with a concomitant decrease in the percentage of cells in the G_2_ phase. These effects appear to be mediated by a COX‐independent mechanism involving an increase in p21^Waf1^ and underphosphorylated retinoblastoma (hypo‐pRb1) protein levels. These findings may support a potential role of ASA as adjunctive therapeutic agent in the clinical management of neuroblastoma.

## 1 INTRODUCTION

Neuroblastoma (NB), an embryonic cancer derived from primitive sympathetic neural precursors, is the most common paediatric tumour. Because of its proliferative potential, resistance to apoptosis and highly heterogeneous biological and clinical behaviour, NB standard treatment requires a combined multimodal approach, including chemotherapy, surgery, bone marrow transplant, radiation and immunotherapy.^1^ However, patients affected by NB generally have a poor prognosis and may develop resistance to conventional therapy^1^, ^2^; therefore, new treatments are needed.

The non‐steroidal anti‐inflammatory drug (NSAID) aspirin (or acetylsalicylic acid, ASA) has been associated with a reduced risk for several cancers.^3^, ^4^, ^5^ In addition, many studies have shown that this drug can prevent cell growth and induce apoptosis^6^, ^7^, ^8^ in different tumour models, including nervous system‐derived cancers.^9^, ^10^, ^11^

Regarding NB, some studies showed that diclofenac, a non‐steroidal anti‐inflammatory drug, is able to induce apoptosis and cell growth inhibition in vitro and in xenografts in vivo.^12^ In addition, ciclooxygenase‐2 (COX‐2) participates in the development and progression of different tumours, including neuroblastoma^13^, ^14^ and COX‐2 overexpression is associated with resistance to apoptosis, induction of metastases and neo‐angiogenesis in NB cell lines.^15^ However, the effects of NSAIDs, and specifically of ASA, on these tumours are still poorly characterized. In the present study, we analysed the effects of ASA on NB cells growth and its putative underlying molecular mechanism(s), by using as a model the SK‐N‐SH (N) cells, a subpopulation of human neuroblastoma SK‐N‐SH cell line. Since neuroblastoma cells retain the ability to undergo neuronal differentiation in the presence of appropriate signals^16^, ^17^ and the induction of the latter is a promising NB treatment approach,^18^ we also used SK‐N‐SH (N) cells to study the effects of ASA on neuronal differentiation.

## 2 MATERIALS AND METHODS

### 2.1 Separation, purification and culture conditions of SK‐N‐SH (N) cells

Human neuroblastoma SK‐N‐SH (ATCC LCG Promochem, Milan, Italy, ATCCR No HTB‐11) cells were cultured in 75 cm^2^ tissue culture flasks in MEM with Earle's salts (Biochrom KG), supplemented with 10% foetal bovine serum (Biochrom KG), 2 mmol/L l‐glutamine, 100 U/mL penicillin, 100 μg/mL streptomycin, non‐essential amino acids and sodium pyruvate. The cells were incubated at 37°C in a humidified atmosphere consisting of 5% CO_2_ and 95% O_2_. The culture medium was changed every 2 days, and cells were allowed to grow until they reached confluence. At this time, the culture medium was replaced with PBS without Ca^2+^ and Mg^2+^ plus antibiotics, and the cells were incubated for 15 minutes at 37°C. The flasks were then gently shaken to separate the weakly adherent neuroblast‐like subpopulation, SK‐N‐SH (N), from the epithelial‐like SK‐N‐SH (E) cells monolayer. The detached SK‐N‐SH (N) cells were collected and re‐cultured in MEM with Earle's salts containing 10% foetal bovine serum, 2 mmol/L l‐glutamine, 100 U/mL penicillin, 100 μg/mL streptomycin, non‐essential amino acids and sodium pyruvate. At confluence, cells were again purified as described earlier. This separation procedure was repeated at least 15 times, until the appearance of the cellular neuroblast‐like phenotype became uniform.^19^

### 2.2 Drug treatment and analysis of cell growth

ASA was purchased from Sigma‐Aldrich (St. Louis, MO, USA). Stock solutions (100 mmol/L) were prepared by dissolving the substance in ethanol (vehicle) and diluted to the desired concentrations in incubation medium. Prostaglandin E_2_ (PGE_2_) was purchased from Sigma‐Aldrich. Stock solutions (5 mmol/L) were prepared by dissolving the substance in ethanol (vehicle) and diluted to the desired concentrations in incubation medium. Cells were cultured in 60 mm Petri dishes (50 000 cells/dish) and treated with 2 mmol/L ASA or vehicle (control). The drug was added to the cultures starting from the second day after plating and thereafter every 48 hours, and growth was evaluated, every 24 hours, by counting the cells in quadruplicate. In experiments with PGE_2_, the latter was added to cell cultures together with ASA. Cell viability was determined by trypan blue dye exclusion.

### 2.3 LDH release assay

Lactate dehydrogenase released in the incubation media was quantified using the CytoTox‐96 cytotoxicity assay kit from Promega, according to the manufacturer's instructions. Plates were incubated at room temperature for 30 min in the dark, and adsorbance was then measured at 490 nm with a microimmunoanalyzer (Labsystems Oy).

### 2.4 Wound healing assay

Cells were cultured in 35 mm Petri dishes to confluence, in order to form a monolayer covering the surface of the entire plate. Wounds were created with a pipette tip as previously described^19^ and washed extensively with MEM to remove cell debris. Cells were then treated with 2 mmol/L ASA or with vehicle. The open gaps were microscopically assessed over time as the cells moved in and filled the damaged area. Images were taken at 6 and 24 hours during cell migration, and the latter was quantified by measuring the distance between two defined points on either side of the gap with the program Image J. For statistical evaluation, at least three measurements at different points were performed at each image.

### 2.5 Microscopic analysis of cell death

The morphological features of apoptotic degeneration were analysed through the use of fluorescence microscopy with the nuclear dye Hoechst 33 258.^19^ Cells were plated at a density of 1 000 000 cells per 35 mm dish containing a glass coverslip. After exposure to the experimental treatment, cells were washed with PBS and fixed in 4% paraformaldehyde in PBS for 30 minutes at room temperature. Cells were then washed three times with PBS and incubated for 10 minutes in a humidified atmosphere of 5% CO_2_, 95% O_2_ at 37°C with the DNA‐binding dye Hoechst 33 258 (0.5 μg/mL in PBS). After being washed with distilled water, coverslips were mounted onto glass slides and photographed on a fluorescence microscope (Nikon Eclipse TE 300) with excitation at 360 nm and magnification of ×400. Cells were considered viable if their chromatin was diffused and evenly distributed throughout the nucleus, whereas cells with bright, condensed and/or fragmented nuclei were counted as positive for apoptosis. Normal and apoptotic cells were counted from three fields per dish in a fixed pattern.

### 2.6 Cell cycle analysis

The distribution of cells within the cell cycle was determined by flow cytometric analysis of DNA content (EPICS XL‐MCL Flow Cytometer; Coulter Electronics). Cells (2 000 000/mL) were washed twice with ice‐cold PBS, resuspended in 50 μl PBS/HBSS containing 2% FBS and fixed at 4°C for 30 minutes with 1 mL 80% ice‐cold ethanol. Before use, cells were washed three times at 4°C, incubated with 0.5 mL PBS and 0.5 mL DNA‐Prep stain (Coulter Reagents) containing RNase (4 kU/mL) and propidium iodide (50 µg/mL) and maintained at room temperature for 30 minutes in the dark. DNA histograms were analysed using Multicycle AV software (Phoenix, San Diego, CA, USA) to evaluate cell cycle distribution.

### 2.7 Western blot analysis

SK‐N‐SH (N) cells (2‐3 000 000 cells per 60 mm dish) were lysed and sonicated with two pulses of 5 seconds with 50% of amplitude (Sonics and Materials, Newton, CT, USA) in a small volume of detergent lysis buffer containing 1% NP‐40, 0.01% SDS, 20 mmol/L Tris‐HCl pH 7.4, 300 mmol/L NaCl, 1 mmol/L EGTA, 1 mmol/L EDTA, 0.1 mmol/L DTT, 1 mmol/L Na_3_VO_4_ and protease inhibitors cocktail (Sigma‐Aldrich). Equal amounts (30‐40 μg) of total protein extracts, determined using Bio‐Rad protein Assay (Bio‐Rad), were loaded on NuPAGE Bis‐Tris gels, (Invitrogen), transferred on Hybond‐C Extramembrane (Amersham Biosciences; GE Healthcare Life Science) and immunoblotted using the following primary antibodies: rabbit anti‐p21 and anti‐p27 (Santa Cruz Biotechnology, Santa Cruz, CA, USA), mouse anti‐underphosphorylated‐Rb1 (BD Pharmingen), rabbit anti‐TH (Cell Signaling, MA, USA), rabbit anti‐Survivin (Abcam) and mouse anti‐β‐actin (Sigma‐Aldrich). After three washes with TBS‐T buffer, immunoreactive proteins were detected using rabbit‐anti‐mouse and donkey‐anti‐rabbit horseradish peroxidase‐conjugated secondary antibodies directed to the appropriated primary antibodies (Jackson Immuno Research Laboratories). The proteins were then visualized using a chemiluminescence system (ECL Millipore). The densitometric analyses of bands normalized to β‐actin protein levels were performed using the ImageMasterR VDS and the Imagesystem software package (Amersham‐Pharmacia Biotech).

### 2.8 Statistical analysis

Data were analysed by one‐way ANOVA, followed by post hoc Newman‐Keul test for multiple comparisons among group means, using a PrismTM software (GraphPad), and differences were considered statistically significant if *P* < .05. All results are presented as the mean ± SEM of at least three different experiments performed in duplicate, unless otherwise specified.

## 3 RESULTS

### 3.1 Effects of ASA on proliferation, cell viability and cell cycle in SK‐N‐SH (N) cells

**Figure 1:**
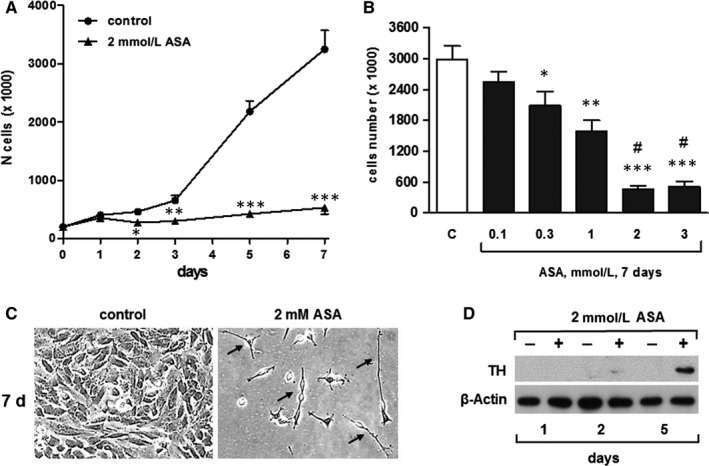
ASA inhibits the proliferation and induces neuronal‐like differentiation of SK‐N‐SH (N) cells in a time‐ and concentration‐dependent manner. A, Time course of the effects of ASA on SK‐N‐SH (N) cells. Cells were incubated with vehicle (control) or with medium containing 2 mmol/L ASA for the indicated times. Results are from three independent experiments performed in duplicate. *P < .05, **P < .01 and ***P < .001 (vs control). B, Concentration dependence of the effects of ASA on SK‐H‐SH (N) cells. Cells were incubated with vehicle or with medium containing increasing concentrations of ASA for 7 d. Results are from three independent experiments performed in duplicate. *P < .05, **P < .01 and ***P < .001 (vs control); #*P* ˂ 0.05 (vs 0.3 mmol/L ASA). C, Light microscopic representative images at ×200 magnification showing ASA‐treated SK‐N‐SH (N) cells vs control, after 7 d. D, Representative Western blot experiment showing the time course of the effects of ASA on tyrosine hydroxylase (TH) in SK‐N‐SH (N) cells

SH‐N‐SH (N) cells were incubated with 2 mmol/L ASA and counted daily, during a 7 days period of culture (Figure 1A). ASA dramatically inhibited cell proliferation, compared with control, in a time‐dependent manner. The concentration dependence of this effect was analysed with a 7‐day exposure of cells to ASA, which showed a maximal effect at 2 mmol/L (Figure 1B).

When observed under a microscope, ASA‐treated cells showed major morphological changes compared with control. In the presence of ASA, cells exhibited small, brilliant, round or spindle‐shaped cell bodies, with thin elongated and branched neurite extensions. On the contrary, the untreated controls retained wide cell bodies with short cytoplasmic processes. These phenotypic changes became apparent within the second day and were entirely developed within 7 days of treatment (Figure 1C). The differentiating effect of ASA was further confirmed by measuring the protein expression of tyrosine hydroxylase (TH), a specific neuronal differentiation marker.^20^ Western blotting analysis showed the appearance of tyrosine hydroxylase at 2 days, with the maximal expression after 5 days in ASA‐treated samples, demonstrating that cells eventually exit from the cell cycle and differentiate at this point (Figure 1D).

**Figure 2:**
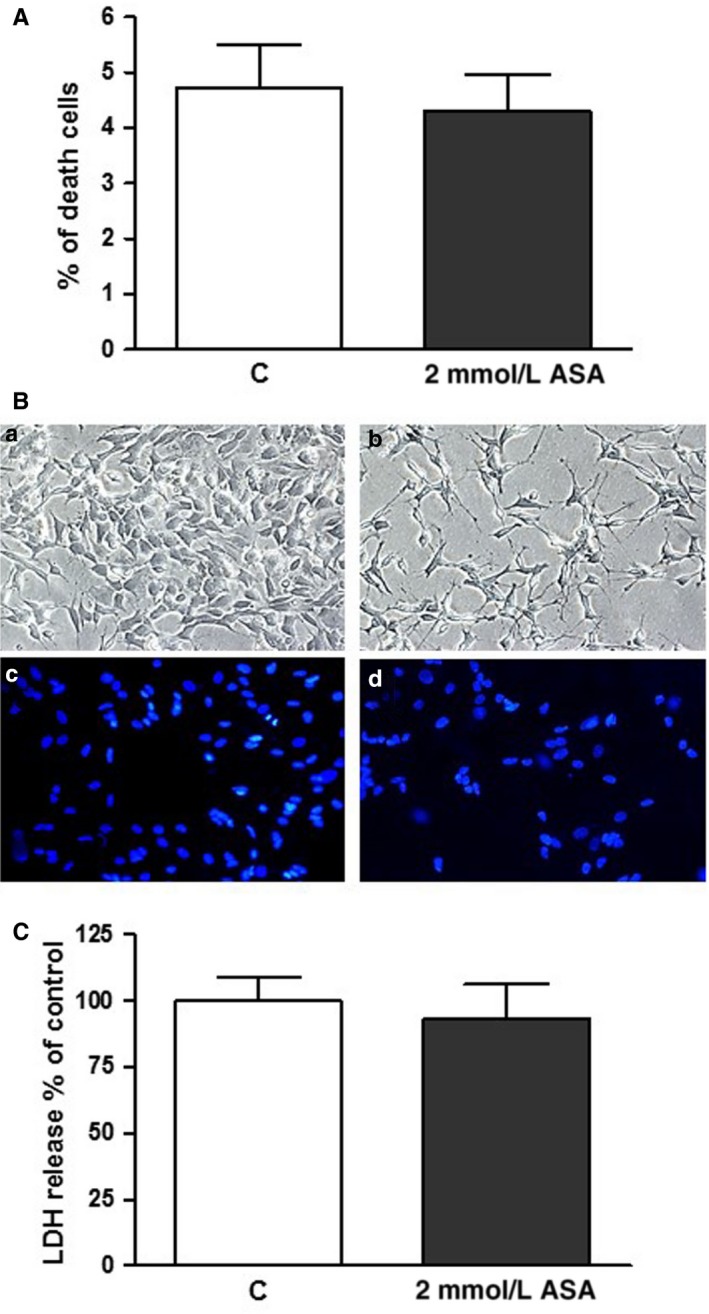
ASA does not affect cell viability of SK‐H‐SH (N) cells. A, Cells were incubated with vehicle (control) or with medium containing 2 mmol/L ASA for 3 d, and then, viability was measured *via* trypan blue exclusion. B, Cells were incubated with vehicle (control) (a) or with medium containing 2 mmol/L ASA (b) for 3 d and photographed in phase contrast, or stained with Hoechst 33 258 for visualization of the nuclei [c, control; d, 2 mmol/L ASA]. C, Cells were incubated with vehicle (control) or with medium containing 2 mmol/L ASA for 3 d, and LDH was measured. Results are from three independent experiments performed in duplicate

In all experiments, over than 95% of the cells were viable in both control and treated conditions (Figure 2A), and there was <5% of dead cells in each group, for up to 7 days of treatment. Therefore, we used the Hoechst 33 258 staining assay to exclude the possibility that ASA induced apoptosis in SK‐N‐SH (N) cells. As expected, we did not observe apoptotic bodies in significant numbers in either ASA‐treated or control cells (Figure 2B). Similarly, we did not detect any changes in lactate dehydrogenase (LDH) release with respect to control (Figure 2C). Thus, incubation with ASA does not induce cell death but instead directly inhibits cell proliferation.

However, ASA induced an accumulation of SK‐N‐SH (N) cells in the G_0_/G_1_ phase of the cell cycle and a decrease in the percentage of cells in the G_2_ phase (Table 1). These results suggest that ASA can induce a G_0_/G_1_ arrest and hence delays cell cycle progression in neuroblastoma cells, exerting a cytostatic, rather than a cytotoxic effect.

**Table 1:** Aspirin causes a G_0_/G_1_ cell cycle arrest in SK‐N‐SH (N) cells

		% G_0_/G_1_	% S	% G_2_
1 d	Control	32.49 ± 2.68	31.43 ± 1.33	36.08 ± 3.98
	2 mmol/L ASA	43.44 ± 1.91*	23.69 ± 3.20	32.87 ± 3.94
4 d	Control	38.73 ± 9.92	26.94 ± 3.25	34.33 ± 3.61
	2 mmol/L ASA	65.95 ± 5.22**	21.74 ± 3.29	12.32 ± 3.48**

Time course of the effects of aspirin on SK‐N‐SH (N) cell cycle. Cells were incubated with vehicle (control) or with 2 mmol/L aspirin for the indicated times. Results are from two independent experiments performed in duplicate.

* *P* < .05;

** *P* < .01 (vs control).

### 3.2 ASA inhibits the proliferation of SK‐N‐SH (N) cells in a COX‐independent manner

**Figure 3:**
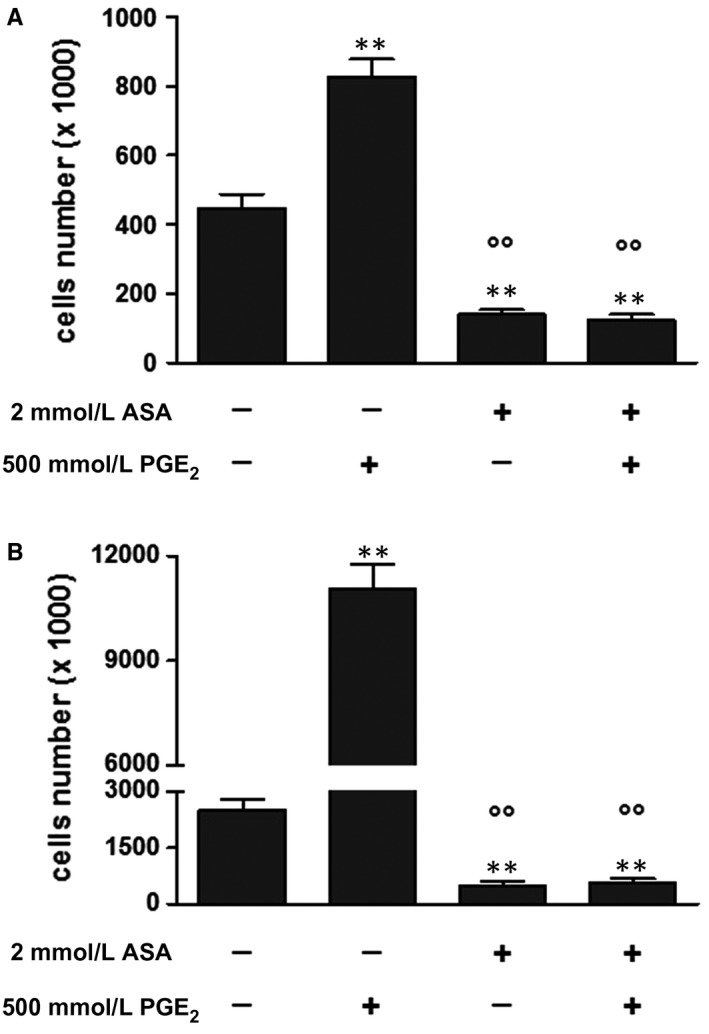
ASA inhibits the proliferation of SK‐H‐SH (N) cells in a COX‐independent manner. A‐B, Cells were incubated with vehicle or with medium containing 2 mmol/L ASA, 500 nmol/L PGE_2_ or both substances for 2 and 5 d. Results are from three independent experiments performed in duplicate. **P < .01 (vs control); °°*P* ˂ 0.01 (vs PGE_2_ alone)

Acetylsalicylic acid is a non‐selective irreversible inhibitor of both COX‐1 and COX‐2.^21^ COX‐2 and PGE_2_ receptors are usually expressed in neuroblastoma cells, and inhibition of COX‐2‐derived PGE_2_ signal results in a modulation of cell growth.^22^ In addition, COX‐1 is constitutively expressed in SK‐N‐SH (N) cells.^23^, ^24^ We investigated whether exogenously added PGE_2_ could rescue SK‐N‐SH (N) from the ASA anti‐proliferative action, by incubating the cells with 500 nM PGE_2_ alone or in the presence of 2 mmol/L ASA. Although PGE_2_ treatment significantly increased cell proliferation in a time‐dependent manner, it did not abrogate ASA‐mediated growth inhibition (Figure 3). Therefore, PGE_2_ may certainly act as a proliferating factor for neuroblastoma cells, but in our experimental model, the effects of ASA were mediated *via* a COX‐independent mechanism.

### 3.3 ASA reduces SK‐N‐SH (N) cells motility

**Figure 4:**
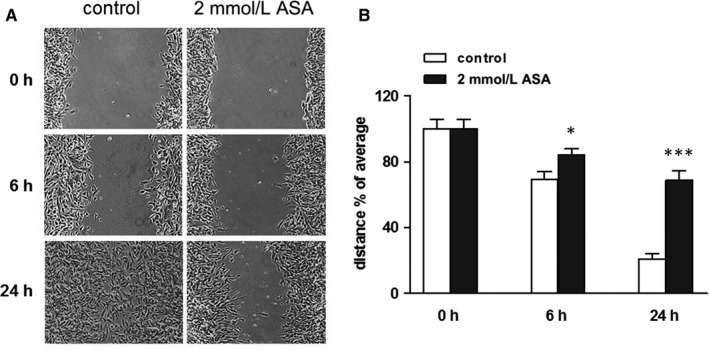
ASA reduces SK‐H‐SH (N) cells motility. A, Effects of 2 mmol/L ASA on cell migration in a wound healing assay. The figure shows a representative experiment. B, Histogram of the percentage of open wound remaining after the indicated times. Results are from three independent experiments performed in duplicate. *P < .05 and **P < .01 (vs control)

An increase in cell motility is crucial for the metastatic potential of tumour cells; therefore, we performed a wound healing assay to investigate the effect of ASA on SK‐N‐SH (N) cells motility. To this purpose, a line was formed by scratching the cell monolayer with a tip (Figure 4A). In this model at the early time‐points, the gap is mainly filled by cells that move into it rather than cells that proliferate, while after 24 h the gap‐filling results from a combination of proliferation and motility. After wounding, cells were incubated with ASA (time 0), and the gap size was measured at different time‐points and compared with control cells. Results are presented as % of the distance that remained open at that particular time‐point. After 6 hours, 69.4% and 84.5% of the initial gap were still open in control and ASA‐treated cells, respectively. At 24 hours, only 21.1% of the gap was still open in control cells, while about 68.9% was uncovered in ASA‐treated cells (Figure 4B), suggesting that ASA reduced the motility of SK‐N‐SH (N) cells.

### 3.4 ASA increases p21^Waf1^ and underphosphorylated retinoblastoma (Rb1) protein levels, and decreases the expression survivin in SK‐N‐SH (N) cells

**Figure 5:**
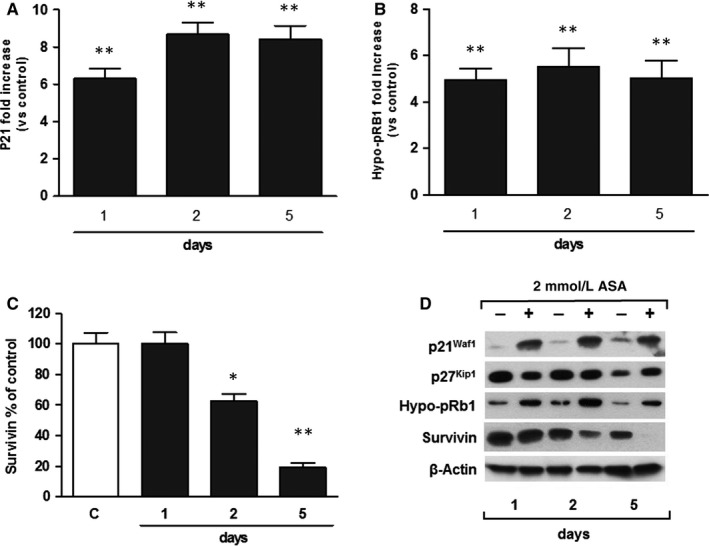
ASA modulates Rb1 pathway *via* p21^Waf1^ up‐regulation and decreases survivin expression in SK‐N‐SH (N) cells. A, Time course of the effects of ASA on p21^Waf1^ protein levels in SK‐N‐SH (N) cells. Cells were incubated with vehicle (control) or with medium containing 2 mmol/L ASA, for the indicated times. Results, expressed as fold increase of protein levels in treated cells vs their respective time‐point controls, are from three independent experiments performed in duplicate. **P < .01 (vs control). B, Time course of the effects of ASA on hypo‐phosphorylated Rb protein levels in SK‐N‐SH (N) cells. Cells were incubated with vehicle (control) or with medium containing 2mM ASA for the indicated times. Results, expressed as fold increase of protein levels in treated cells vs their respective time‐point controls, are from three independent experiments performed in duplicate. **P < .01 (vs control). C, Time course of the effects of ASA on survivin protein levels in SK‐N‐SH (N) cells. Cells were incubated with vehicle (control,) or with medium containing 2mM ASA for the indicated times. Results, expressed as percentage of protein levels in treated cells vs their respective time‐point controls, are from three independent experiments performed in duplicate. *P < .05 and **P < .01 (vs control). D, Representative images of Western blots for p21^Waf1^, p27^Kip1^, hypo‐phosphorylated Rb1 (Hypo‐pRb1) and survivin

Since aspirin inhibits growth and causes G_0_/G_1_ cell cycle arrest in SK‐N‐SH (N) cells, we studied its effects on the expression of the cell cycle‐related proteins p21^Waf1^ and p27^Kip1^ that are highly expressed in these cells.^17^ ASA rapidly and selectively increased the endogenous protein levels of p21^Waf1^ in a time‐dependent manner. p21^Waf1^ significantly increased within 1 day, peaked after 2 days and remained high for at least 5 days of treatment (Figure 5), suggesting a direct involvement of this protein in the G_0_/G_1_ cell cycle arrest observed in SK‐N‐SH (N) cells. On the contrary, p27^Kip1^ was not affected by the treatment with ASA.

**Figure 6:**
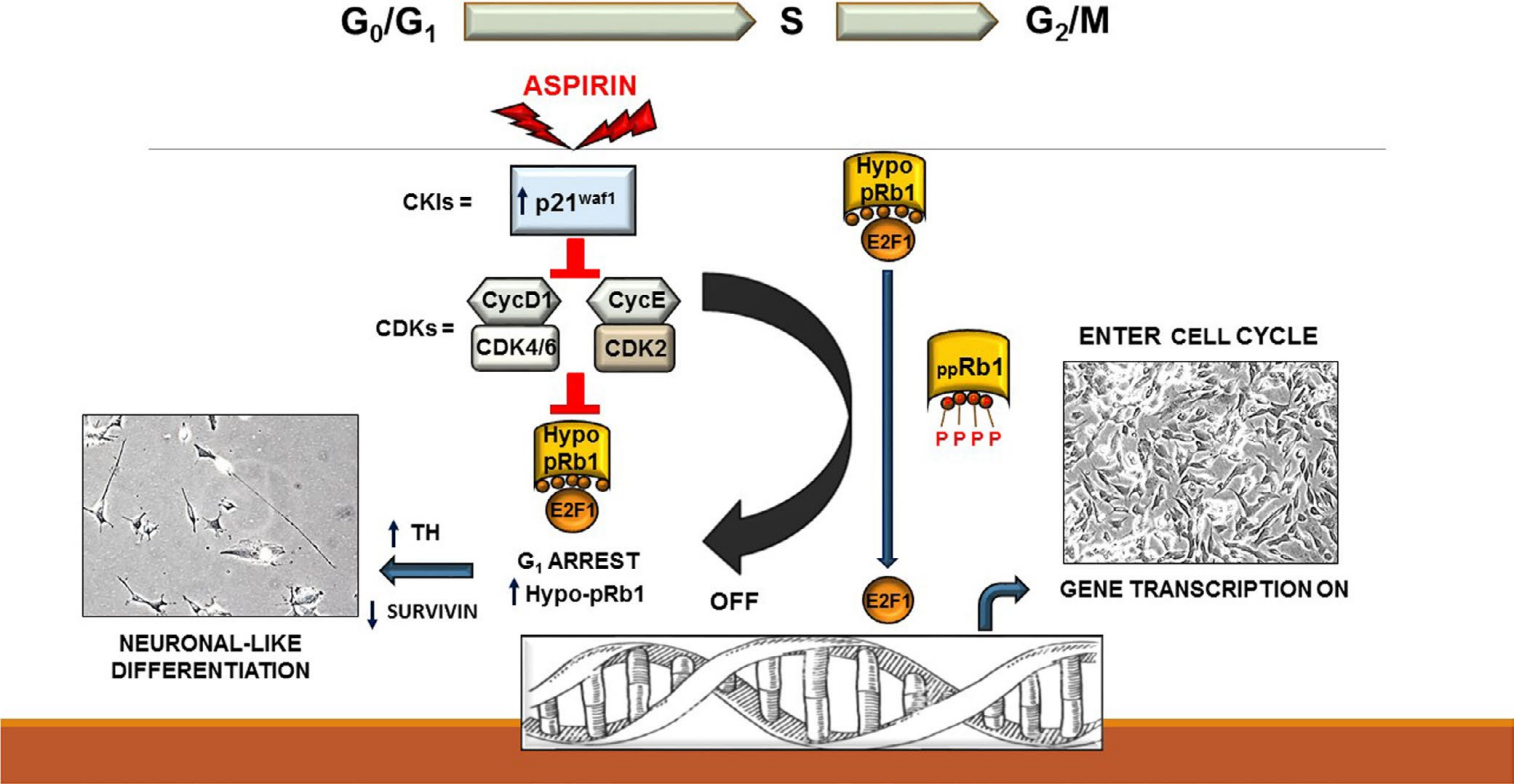
Schematic model of the effects of ASA in SK‐N‐SH (N) cells. ASA induces an up‐regulation of CKI p21^Waf1^ and the accumulation of Rb1 protein in its hypo‐phosphorylated form (Hypo‐pRb1). The latter may bind and inhibit E2F1 transcriptional activity. Following this effect, SK‐H‐SH (N) cells undergo G_0_/G_1 _cell cycle arrest and move towards the neuronal‐like differentiation, inducing the expression of tyrosine hydroxylase (TH) and decreasing the levels of survivin

p21^Waf1^ accumulation might impair CDK2 and CDK4 functions, which promote G_1_‐to‐S transition by phosphorylating the retinoblastoma (Rb1) protein^25^; therefore, we exposed the SK‐N‐SH (N) cells to ASA and analysed its effects on Rb1, by using a specific antibody, which recognizes the underphosphorylated form of this protein (hypo‐pRb1). As shown in Figure 5, ASA produced a significant up‐regulation of hypo‐pRb1 levels after 1 day. These results suggest that Rb1 pathway may be directly involved in the G_0_/G_1_ cell cycle arrest in neuroblastoma cells induced by aspirin (Figure 6).

Interestingly, we found that ASA significantly decreased the amounts of the anti‐apoptotic protein survivin in a time‐dependent manner (Figure 5). This protein, highly expressed in tumour cells, is known to confer resistance to anti‐cancer treatments^26^ and is considered a negative prognostic factor in neuroblastoma.^27^

## 4 DISCUSSION

There is considerable evidence that ASA exhibits powerful anti‐cancer properties. In fact, an increasing number of observational, epidemiological and clinical studies have demonstrated that prolonged, daily use of ASA correlates with a reduced risk for different types of cancer. In addition, ASA can prevent cell growth, *via* COX‐dependent and COX‐independent mechanisms, in several tumour models in vitro and in vivo.^28^, ^29^, ^30^, ^31^

Neuroblastoma (NB), a paediatric cancer derived from primordial neural crest precursors, is the most common extracranial solid tumour of childhood. Because of its proliferative potential, resistance to apoptosis and high biological heterogeneity, which accounts for variable clinical behaviour, NB standard treatment requires a combined multimodal approach, including chemotherapy, surgery, radio‐ and immunotherapy, as well as bone marrow transplant.^1^ In some cases, the tumour may regress completely, or spontaneously differentiate, but generally, patients affected by NB have a poor prognosis and may develop resistance to conventional therapy.^1^, ^2^ Indeed, the long‐term survival rates for patients with high‐risk NB are currently <50% despite the aggressive therapy, emphasizing the need to find new treatments.^32^

Here, we used the SK‐N‐SH (N) cells, a subpopulation of human neuroblastoma SK‐N‐SH cell line, as a model to investigate the effects of ASA on NB cells proliferation as well as the putative underlying molecular mechanism(s). In this experimental model, ASA strongly inhibited cell proliferation in a time‐ and concentration‐dependent manner. The maximal effect was obtained at a dose of 2 mmol/L, which falls within the physiological relevant concentrations of salicylic acid (0.5–2.5 mmol/L), normally found in the plasma with analgesic/anti‐inflammatory ASA dose,^5^ and is also highly effective in inhibiting glioblastoma multiforme (GBM) stem cells growth in vitro.^11^ In addition, ASA dramatically changed SK‐N‐SH (N) cells morphology, showing differentiation into a more mature neuronal phenotype. This observation was confirmed by the induction of tyrosine hydroxylase protein expression, a marker of neuronal differentiation specific for this cell line.^20^ The morphological changes were similar to those observed after treatment with retinoic acid, a well‐known neuroblastoma differentiating agent^17^ [Pozzoli G. and Cenciarelli C., personal observation], suggesting that ASA might force neuroblastoma cells to exit cell cycle and to differentiate towards the neuronal‐like phenotype. Although previous studies reported that aspirin generally induces apoptosis in nervous system‐derived cancers *via* the inhibition of SHH/GLI1 or IL‐6/STAT3 signalling pathways,^7^, ^31^ it has been shown that the drug might also act as a differentiating agent in other types of neoplastic cells.^33^ In addition, ASA differentiating actions have been demonstrated in several physio‐pathological conditions, like osteogenic differentiation of human dental stem cells,^34^ oligodendrocytes differentiation following white matter lesion^35^ or cardiomyocytes differentiation of bone marrows mesenchymal stem cells.^36^

Since ASA may affect cancer proliferation either through COX‐dependent and/or COX‐independent mechanisms,^10^ we incubated the SK‐N‐SH (N) cells with exogenous PGE_2_ in the presence of ASA. PGE_2_ alone significantly increased cell proliferation, but did not revert ASA‐mediated growth inhibition. Therefore, although PGE_2_ may act as an important proliferating factor for neuroblastoma cells and COX inhibition may be involved in the anti‐proliferative actions of ASA, in our experimental model the drug operated *via* a direct, COX‐independent mechanism. This result is consistent with the effects of ASA observed in glioblastoma cancer stem cells,^11^ as well as in many other tumour models.^28^

We also found that ASA reduced SK‐N‐SH (N) cells motility, which is a key feature of a tumour propagation potential. It should be noted that the scratch test usually measures both cell replication and growth, but these phenomena may be relevant only in the late‐phase of experiments, whereas within the first 6 hours the observed effects are generally related to motility alone.^37^

Acetylsalicylic acid did not cause cell death, but produced an accumulation of cells in the G_0_/G_1_ phase of the cell cycle, with a parallel decrease of cells in G_2_ phase.

The cyclin‐dependent kinases (CDKs), and their antagonists, the CDK inhibitors (CKIs), are crucial factors ensuring the correct cell progression through the cell cycle. Increased expression of one or more CKIs correlates with the accumulation of cells in the G_0_/G_1_ phase. CKIs are grouped into two distinct families: the INK proteins, including p15^Ink4b^, p16^Ink4a^, p18^Ink4c^ and p19^Ink4d^, and the CIP/KIP proteins, which include p21^Waf1^, p27^Kip1^ and p57^Kip2^.^38^ Interestingly, CKIs are also potent tumour suppressor agents^38^; p18^Ink4c^, p21^Waf1^ and p27^Kip1^, for instance, can induce cell cycle arrest and differentiation of many neoplastic cell lines.^39^, ^40^, ^41^ Since p21^Waf1^ and p27^Kip1^ are highly expressed in SK‐NSH (N) cells which, conversely, do not express p15^Ink4b^, p16^Ink4a^, p18 ^Ink4c^ and p19^Ink4d^ mRNAs,^17^ we investigated the effects of ASA on the expression patterns of these proteins. We found that ASA greatly and selectively increased the p21^Waf1^ levels. Downstream, we also observed an accumulation of the Rb1 tumour suppressor protein in its hypo‐phosphorylated form (hypo‐pRb1). Phosphorylated Rb1 predominates in rapidly proliferating cells, while the hypo‐phosphorylated form blocks the progression from G_1_ to S phase and directs the cell towards differentiation (Figure 6).^42^, ^43^, ^44^, ^45^ Interestingly, the retinoblastoma family of proteins plays a central role not only on cell cycle regulation but has other relevant actions, that is regulation of apoptosis, cellular differentiation, preservation of genome stability and ultimately the cell fate.^46^ Apart from p21^waf1^, other CKIs are generally involved in cell cycle control through the modulation of the Rb pathway. In particular, p16^Ink4a^ is a negative regulator of mammalian cell cycle in late G_1_ phase and can induce repression of pRb1 synthesis.^47^ However, in human cells, p16^Ink4a^ seems to be mainly associated with the progression towards the senescent phenotype,^48^, ^49^, ^50^ whereas p21^waf1^ is specifically up‐regulated in cells undergoing neuronal‐like differentiation, including NB cells.^51^, ^52^ Consistently, not only SK‐N‐SH (N) do not constitutively express p16^Ink4a^, or other CKIs of the INK family, but the latter are not induced even when these cells are treated with differentiating agents.^53^ Therefore, although we cannot exclude the possible involvement of other CKIs, we hypothesize that the concomitant p21^waf1^ and hypo‐pRb1 up‐regulation induced by ASA may explain, at least in part, the restraint of the cell cycle entry, indicated by the accumulation of cells in the G_0_/G_1_ phase, and the start of neuronal‐like differentiation of SK‐N‐SH (N) cells (Figure 6).

The inhibitor of apoptosis protein (IAP) survivin is highly up‐regulated in many transformed cell lines including lung, colon, breast and brain human cancers,^54^ where it promotes proliferation, invasiveness and inhibition of apoptosis, the latter in particular conferring resistance to chemotherapy. Therefore, elevated levels of this protein correlate with poor prognosis and radio/chemo‐resistance of several cancers, including NB.^27^, ^55^, ^56^ Here, we report that aspirin time dependently decreased the survivin expression, whose levels almost disappeared after 5 days. Evidence reports that free E2F activators, such as E2F1 and E2F3, directly induce the survivin promoter activity. On the contrary, the hypo‐phosphorylated Rb1 protein, preventing E2F release, would favour the interaction of E2F repressors (E2F4 and E2F5) on the promoter of survivin, allowing for a gene transcription repression in non‐transformed embryonic fibroblast cells.^57^ Our data are suggestive for linking hypo‐pRb1 to the negative regulation of survivin protein expression in SK‐H‐SH (N) cells.^57^ The inhibition of survivin may represent an additional advantage in neoplastic patients, since through this action aspirin might enhance the effect of cytotoxic drugs to cancer cells.

In conclusion, here we show that ASA greatly inhibits neuroblastoma cells proliferation and motility, and promotes their differentiation in vitro. These effects are mediated, at least in part, by the increase in p21^Waf1^ levels, associated with an accumulation of hypo‐pRb1, suggesting that ASA might be potentially useful as an adjuvant therapy in the treatment of NB patients. However, this potential does not take in account the side effects of ASA; therefore, the use of this compound as an adjuvant therapy, especially for paediatric patients, would require caution.^58^ Moreover, a very recent study, while confirming that the chronic use of ASA correlates with a reduced risk for different cancers, has shown that, unexpectedly, it could also have opposite effects, in particular by increasing the risk of breast cancer.^59^ In this light, further in vivo preclinical investigation, possibly involving human tumour xenograft of NB in nude mice, will be required, in order to confirm the efficacy of ASA and to consider a careful risk‐benefit assessment.

## CONSENT FOR PUBLICATION

Approved by all authors.

## CONFLICT OF INTEREST

The authors declare that there is no conflict of interest.

## AUTHOR CONTRIBUTIONS

GP and CC conceived the study, supervised the experiments, collected the data, conceived the paper and wrote the primary draft. CC contributed to execution of Western blots. GP contributed to the execution of cell growth and biological assays. G.Pe. contributed to execution of PGE_2_ treatment on neuroblastoma cells. HEM contributed to statistical analysis and revised the entire draft. PN contributed to the final manuscript editing. All authors analysed the data, read and approved the final manuscript.
